# The genetic study utility of a hexaploid wheat DH population with non-recombinant A- and B-genomes

**DOI:** 10.1186/2193-1801-2-131

**Published:** 2013-03-25

**Authors:** Ming Hao, Jixiang Chen, Lianquan Zhang, Jiangtao Luo, Zhongwei Yuan, Zehong Yan, Bo Zhang, Wenjie Chen, Yuming Wei, Huaigang Zhang, Youliang Zheng, Dengcai Liu

**Affiliations:** 1 Triticeae Research Institute, Sichuan Agricultural University, Wenjiang, Chengdu, 611130 Sichuan P.R. China; 2 Key Laboratory of Adaptation and Evolution of Plateau Biota, Northwest Institute of Plateau Biology, Chinese Academy of Sciences, Xining, 810001 P.R. China

**Keywords:** Allopolyploid, Aegilops tauschii, Doubled-haploid, Segregation distortion

## Abstract

To study the D-genome of the wild wheat relative *Aegilops tauschii* Cosson at the hexaploid level, we developed a synthetic doubled-haploid (DH) hexaploid wheat population, SynDH3. This population was derived from the spontaneous chromosome doubling of triploid F_1_ hybrid plants obtained from a cross between *Triticum turgidum* ssp. *dicoccon* PI377655 and *A. tauschii* ssp. *strangulata* AS66 × ssp. *tauschii* AS87. SynDH3 is a diploidization-hexaploid DH population containing recombinant D chromosomes from two different *A. tauschii* genotypes, with A and B chromosomes from *T. turgidum* being homogenous across the entire population. Using this population, we constructed a genetic map. Of the 440 markers used to construct the map, 421 (96%) were assigned to 12 linkage groups; these included 346 Diversity Arrays Technology (DArT) and 75 simple sequence repeat (SSR) markers. The total map length of the seven D chromosomes spanned 916.27 cM, with an average length of 130.90 cM per chromosome and an average distance between markers of 3.47 cM. Seven segregation distortion regions were detected on seven linkage groups. Out of 50 markers shared with those on a common wheat map, 37 showed a consistent order. The utility of the diploidization-hexaploid DH population for mapping qualitative trait genes was confirmed using the dominant glaucousness-inhibiting gene *W2*^*I*^ as an example.

**Electronic supplementary material:**

The online version of this article (doi:10.1186/2193-1801-2-131) contains supplementary material, which is available to authorized users.

## Introduction

Common wheat, or bread wheat (*Triticum aestivum* L., 2*n* = 6*x* = 42, AABBDD), arose from the hybridization of *T. turgidum* L. (2*n* = 4*x* = 28, AABB) with the wild wheat relative *Aegilops tauschii* Cosson (2*n* = 2*x* = 14, DD). Because of an evolutionary bottleneck, genetic diversity within the D-genome of *A. tauschii* is much higher than within the D-genome of common wheat, as most *A*. *tauschii* populations were not involved in common wheat speciation. Although *A. tauschii* has been used for improvement of common wheat (Reif et al. 2005; Warburton et al. 2006; Van Ginkel and Ogbonnaya 2007; Yang et al. 2009; Li et al. 2011; Reynolds et al. 2011), most of its genetic potential, especially with respect to quantitative trait loci (QTL) controlling economically important traits such as yield, flour quality, and stress tolerance, remains unexploited.

Genetic mapping with molecular markers capable of tracking introduced genomic regions is an important tool for improving the transfer efficiency of alien traits. Genetic maps, based on amplified fragment length polymorphism (AFLP), restriction fragment length polymorphism (RFLP), microsatellite (SSR), and/or single-nucleotide polymorphism (SNP) markers, have been constructed for the *A. tauschii* D-genome using segregating populations derived from hybrids of *A. tauschii* accessions (Gill et al. 1991; Boyko et al. 1999; Ter Steege et al. 2005; Luo et al. 2009). Diversity Arrays Technology (DArT), which uses microarray hybridization to detect the presence or absence of DNA fragments, is a highly effective genetic mapping technology (Jaccoud et al. 2001; Wenzl et al. 2004; Akbari et al. 2006). It has not been used for mapping of *A. tauschii*, however.

*Aegilops tauschii* is diploid, whereas bread wheat is hexaploid. Gene expression can be greatly altered by different ploidy levels and/or the roles of homoeologous genes (Qi et al. 2012and cited references). To exploit the genetic potential of *A. tauschii* for wheat improvement, genetic analysis at the hexaploid level is consequently important. Construction of a D-genome genetic map is much more complicated for hexaploid wheat than for diploid *A. tauschii*, however, because many homoeologous (highly similar but non-allelic) sequences are present in the homoeologous A- and B-genomes of hexaploid wheat. Distinguishing homoeologous from homologous markers is complicated and prone to error (Poole et al. 2007; Barker and Edwards 2009; Allen et al. 2011). To reduce this complexity, we developed a synthetic doubled-haploid (DH) population (SynDH) in a hexaploid background (Zhang et al. 2011; Luo et al. 2012) consisting of genetically-recombined D-genome chromosomes from two *A. tauschii* accessions under a background of non-recombinant A- and B-genomes from a *T. turgidum* line. This diploidization-hexaploid SynDH population thus differed from current hexaploid wheat populations in which the A-, B-, and D-genomes are all involved in recombination. Our goal was to evaluate the usefulness of a SynDH population in genetic studies by (i) developing a genetic map of the *A. tauschii* genome using DArT and SSR markers, (ii) comparing this map with previously-reported ones, (iii) assessing the presence and extent of segregation distortion, and (iv) mapping the gene for glaucousness, as an example of a qualitative trait gene.

## Materials and methods

### Plant materials

Plant materials used in this study included 39 lines of a DH population and its three parents, *T. turgidum* ssp. dicoccon PI377655, *A. tauschii* ssp. *tauschii* AS87, and *A. tauschii* ssp. *strangulata* AS66. *Aegilops tauschii* AS87 and *T. turgidum* PI377655 are glaucous, with spike and leaf sheath surfaces coated with a waxy whitish substance, whereas *A. tauschii* AS66 is non-glaucous. *Triticum turgidum* PI377655 was pollinated with pollen from diploid F_1_ hybrids of *A. tauschii* AS66 × AS87 to form triploid F_1_ hybrids with ABD genomes. After selfing of the triploid F_1_ hybrid plants, DH lines were obtained by spontaneous chromosome doubling via union of unreduced gametes (Luo et al. 2012).

### SSR and DArT genotyping

DNA was isolated from bulk leaf samples from five plants for each DH and parental line using the 2×CTAB method (Saghai-Maroofet et al. 1984). A total of 258 SSR markers (http://wheat.pw.usda.gov/cgi-bin/graingenes/browse.cgi?class=marker) were screened for polymorphism in the parents. PCR amplifications and identification of amplified SSR fragments were performed as described in Luo et al. (2012).

Genomic DNA profiling of SynDH lines and parents was carried out using DArT with a common wheat *PstI* (*TaqI*) v3.0 DArT array by Triticarte (Canberra, Australia; http://www.triticarte.com.au/). For each sample, each marker was scored as “1” (present), “0” (absent), or, if it could not be reliably scored for that sample, as “-” (missing). DArT calls were converted into “A” (AS66), “B” (AS87), and “-” (missing data) by comparison against parental scores.

### Map construction

Map construction and comparison were carried out as described in Zhang et al. (2012), and segregation data were analyzed using QTL IciMapping v3.1 (Li et al. 2007; http://www.isbreeding.net/software/?type=detail&id=3). Markers were classified into linkage groups based on a logarithm of odds (LOD) score threshold of 4.7. Markers within each group were then ordered using RECORD (Van Os et al. 2005) and marker order verified using the RIPPLE command with the SARF (sum of adjacent recombination frequencies) option. Graphical genotypes were examined in Excel 2003 (Additional file 1: Table S1). At this step, singletons (single loci in a progeny line that appear to have recombined with both directly-neighboring loci) were replaced by missing values in the data set, and calculations were repeated until no singletons were found. Ungrouped markers (uncorrected data) were anchored to previous linkage groups by using an LOD > 2.0. All calculations were repeated for new linkage groups. Independent linkage groups on the same chromosome with Kosambi distances between subsequent markers less than 50 cM were integrated as one linkage group. The χ^2^ analysis, map drawing, and map comparison were performed using JoinMap 4.0 (Van Ooijen 2006). A χ^2^ goodness-of-fit analysis was performed for each marker to test for deviation from expected 1:1 segregation ratios in the doubled haploids at a significance level of *P* < 0.05. Any region with at least three adjacent loci showing significant segregation distortion was defined as a segregation distortion region (SDR) (Paillard et al. 2003).

## Results

### Genetic map construction

**Figure 1:**
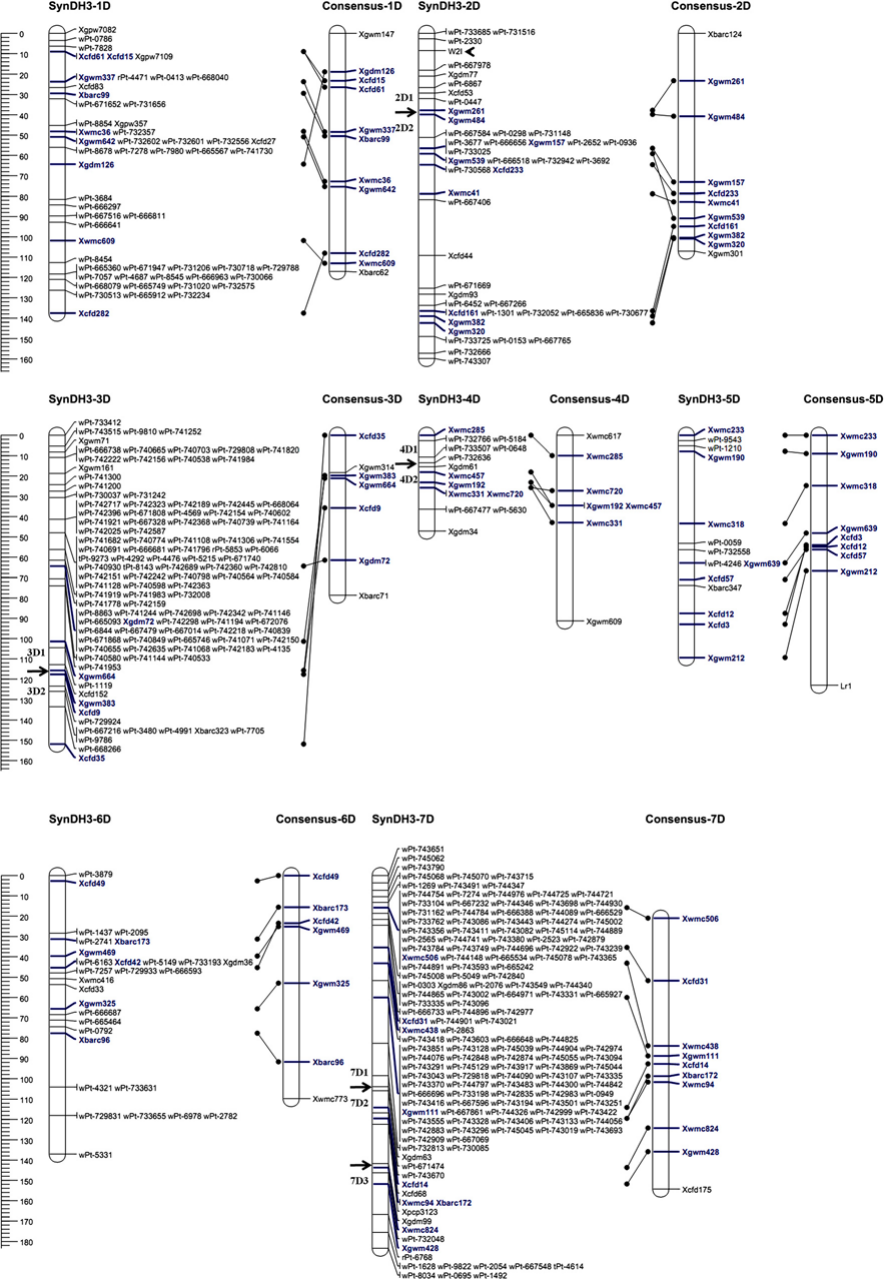
**SynDH3 map obtained in this study and its comparison with a common wheat consensus map (Somers et al.**^2004^**).** Shared markers between the two maps are indicated in blue. Map comparisons were performed using JoinMap 4.0 (Van Ooijen, 2006). The scale on the left indicates distances in cM (Kosambi distances). The arrowheads indicate demarcation zones between two linkage groups.

Because the only recombinant chromosomes in the DH population were those involving D-genomes derived from *A. tauschii* accessions AS66 and AS87, markers showing polymorphism between the two *A. tauschii* parents were used to genotype the DH population for genetic map construction. A set of 440 polymorphic markers, consisting of 79 SSR and 361 DArT markers, was obtained. Of these, 19 (4.3%) were removed from the data set during map construction because anchor markers were lacking. The remaining 421 markers (75 SSRs and 346 DArTs) were successfully mapped onto the final map, forming 12 linkage groups (Additional file 1: Table S1; Figure 1). Based on the shared marks between our map with previous reported D-genome maps for common wheat (Somers et al. 2004), the 12 linkage groups were assigned to 1D-7D chromosomes, respectively. Each D chromosome contained one to three linkage groups (Table 1). The order of multiple linkage groups on same chromosome were decided according to the shared markers in common wheat consensus map (Figure 1). The total map length of the seven D chromosomes spanned 916.27 cM, with an average length of 130.90 cM per chromosome and an average distance between markers of 3.47 cM. Reme¿ and Matysioková

**Table 1:** **Marker distribution on D-genome chromosomes**

Chromosome	No. of linkage groups	Length (cM)	Number of target markers					Average distance between markers (cM)	Mean density**
			Total	SSR	DArT*				
					wPt	rPt	tPt		
1D	1	137.61	54	14	39	1	-	2.55	6.26
2D	2	157.84	44	13	31	-	-	3.51	6.31
3D	2	149.93	112	9	100	1	2	1.34	6.52
4D	2	45.10	14	7	7	-	-	3.22	5.01
5D	1	109.38	14	9	5	-	-	7.81	9.12
6D	1	137.08	29	9	20	-	-	4.73	8.57
7D	3	179.33	154	14	138	1	1	1.16	6.64
Total	12	916.27	422	75	340	3	3	3.47	6.92

* wPt, rPt, and tPt indicate markers derived from wheat, rye, and triticale, respectively.

** equal to L/(*n*-1), where *n* is the number of unique markers per chromosome length L.

### Segregation distortion

Out of 421 mapped markers, 48 (11.4%), including 35 DArT (10.1%) and 13 SSR (17.3%) markers, were significantly distorted (*P* < 0.05) from expected Mendelian segregation ratios. Segregation distortion (SD) was observed on five chromosomes (Additional file 1: Table S1), with frequencies of 9.3% (5 of 54 markers) for chromosome 1D, 13.3% (6/45) for 2D, 10.7% (12/112) for 3D, 24.1% (7/29) for 6D, and 11.7% (18/154) for 7D. In this study, a biological segregation distortion region (SDR) was defined as any region that included at least three uninterrupted markers showing SD. Based on this criterion, seven SDRs were detected within seven linkage groups (Table 2). The SDR on chromosome 1D favored *A. tauschii* AS87 alleles, whereas the six SDRs on the other four chromosomes were skewed toward AS66 alleles (Additional file 1: Table S1).

**Table 2:** **Segregation distortion regions (SDRs) in the SynDH3 population**

Chromosome	Linkage group	Location	No. of markers in linkage group	No. of distorted markers	SDR name	Parental skew
1D	1D	81.58–92.84	5	5	Qsd.scau-1D	AS87
2D	2D2	18.03–37.78	6	6	Qsd.scau-2D	AS66
3D	3D1	5.28–10.77	12	9	Qsd.scau-3D	AS66
6D	6D	65.57–74.37	4	4	Qsd.scau-6D	AS66
7D	7D1	0–7.16	3	3	QSd.scau-7D1	AS66
	7D1	35.38–51.55	9	9	QSd.scau-7D2	AS66
	7D2	0–16.46	6	6	QSd.scau-7D3	AS66

### Map comparisons

To evaluate the quality of the genetic map developed in this study, 50 markers were identified that also appeared on a common wheat consensus map (Somers et al. 2004). The orders of these shared markers were compared between the two maps (Figure 1). Among them, 37 (74%) showed a consistent order on chromosomes 1D (6), 2D (7), 3D (2), 4D (3), 5D (6), 6D (4), and 7D (9). Differences in marker order, including reversed order, were also detected; these discrepancies were found on all chromosomes except for 7D.

### Gene identification

The glaucousness character was surveyed in DH and parental plants during the heading stage. *Aegilops tauschii* AS87 and *T. turgidum* PI377655 were glaucous, whereas *A. tauschii* AS66 was non-glaucous. AS66 × AS87 F_1_ hybrids were also non-glaucous, indicating that the glaucousness of AS87 was inhibited by the epistatic influence of the dominant inhibitor gene in AS66. *W2*^*I*^, the gene for this trait, was further mapped to the distal region of 2DS, and was linked to the DArT marker wPt-2330 within 5.91 cM (Figure 1).

## Discussion

Genetic maps of the *A. tauschii* D-genome have previously been constructed using segregating populations in diploid backgrounds (Gill et al. 1991; Boyko et al. 1999; Ter Steege et al. 2005; Luo et al. 2009). In contrast, our map of the D-genome of *A. tauschii* was constructed in a hexaploid background. Although the DH population used in this study was small—only 39 lines, 96% of 440 polymorphic DNA markers were mapped onto the final *A. tauschii* D-genome map. This high genetic map construction efficiency may be a consequence of the unique genetic structure of the hexaploid DH population used in this study, in which only D-genome chromosomes between *A. tauschii* accessions AS66 and AS87 were involved in genetic recombination under a background of non-recombinant A- and B-genomes from the *T. turgidum* line PI377655 (Luo et al. 2012). Interference due to A- and B-genome polymorphism was thus avoided (Poole et al. 2007; Barker and Edwards 2009; Allen et al. 2011). When 50 shared markers were compared between this map and a consensus map constructed by Somers et al. (2004), 37 exhibited consistent orders. The orders of the remaining 13 differed, however, perhaps as a result of small structural rearrangements (such as translocations, deletions, and inversions) and/or because of the small number of DH lines used.

To evaluate the usefulness of the hexaploid wheat DH population for gene identification in *A. tauschii*, we analyzed the morphological trait of glaucousness. Glaucousness in *A. tauschii* is controlled by a dominant gene, *W2,* located on chromosome arm 2DS*.* This phenotype is inhibited by the epistatic influence of the dominant inhibitor gene *W2*^*I*^ found on the distal region of 2DS (Watanabe et al. 2005; Liu et al. 2007). In our study, the non-glaucous trait was also mapped to 2DS. This result suggests that the diploidization-hexaploid DH population has value as a tool for mapping qualitative trait genes.

Segregation distortion is a common phenomenon in plants and can be influenced by various factors affecting the fertility of either gametes or zygotes (Lyttle 1991). In a previous study on 54 F_2_ diploid plants derived from two *A. tauschii* accessions (Faris et al. 1998), 57 (29%) out of 194 RFLP markers were significantly distorted (*P* < 0.05) from expected segregation ratios, with segregation distortion regions (SDRs) detected on chromosomes 1D, 3D, 4D, 5D, and 7D. In the present study, 48 (11.4%) out of 422 markers showed distorted segregation (*P <* 0.05), and seven SDRs were detected on chromosomes 1D, 2D, 3D, 6D, and 7D. The longest SDR was QSd.scau-7D3, with a length of 16.46 cM including the centromere, and favoring the *A. tauschii* parent AS66 (Additional file 1: Table S1). There may be an important locus associated with this SDR, as a similar SDR has also been detected on a homoeologous chromosome, 7E, in another species (Cai et al. 2011). Out of seven SDRs, six skewed in favor of *A. tauschii* AS66, the maternal parent in the cross with *A. tauschii* AS87. This is consistent with the observations of Faris et al. (1998) that loci affecting gametophyte competition in male gametes via nucleo-cytoplasmic interactions may play a role in SDR production. Allelic variation associated with the production of wide-hybrid plants may have also contributed to the segregation distortion observed in our study, as these hexaploid DH lines were derived from the wide hybridization of *T. turgidum* PI377655 with diploid F_1_ hybrids of *A. tauschii* AS66 × AS87*.* For example, allelic variations in loci that control crossability between different species can affect seed-setting of interspecific crosses (Tixier et al. 1998). Allelic variations related to hybrid seed germination and plant vigor may also affect the production of DH lines (Ter Steege et al. 2005). During the production of the DH lines used in our study, only 16.5% (71/430) of F_1_ hybrid seeds germinated; from these seeds, 39 vigorous haploid plants possessing ABD genomes were obtained (Luo et al. 2012).

We demonstrated in this study that diploidization-hexaploid DH population can be used to generate genetic maps. However, the limitation of a small DH population with only 39 lines should be pointed out. The present study identified 12 linkage groups which were significantly larger than the number of the 7 haploid chromosomes of D genome. The small population could result in more linkage groups and could be one of factors for segregation distortion and inconsistent marker order. To generate a better genetic map, a larger size of mapping population is needed.

## Electronic supplementary material

Additional file 1: Table S1: Marker information used for linkage group construction. (XLS 286 KB)
